# Plasma Vitamin C Concentrations Were Negatively Associated with Tingling, Prickling or Pins and Needles Sensation in Patients with Postherpetic Neuralgia

**DOI:** 10.3390/nu12082384

**Published:** 2020-08-09

**Authors:** Li-Kai Wang, Yao-Tsung Lin, Kuo-Chuan Hung, Chia-Yu Chang, Zhi-Fu Wu, Miao-Lin Hu, Jen-Yin Chen

**Affiliations:** 1 Department of Anesthesiology, Chi Mei Medical Center, Tainan 71004, Taiwan; anesth@gmail.com (L.-K.W.); anekevin@hotmail.com (Y.-T.L.); ed102605@gmail.com (K.-C.H.); aneswu@gmail.com (Z.-F.W.); 2 Department of Health and Nutrition, Chia Nan University of Pharmacy and Science, Tainan 71710, Taiwan; 3 Center of General Education, Chia Nan University of Pharmacy and Science, Tainan 71710, Taiwan; 4 Department of Neurology, Chi Mei Medical Center, Tainan 71004, Taiwan; chiayu.chang7@msa.hinet.net; 5 Center for General Education, Southern Taiwan University of Science and Technology, Tainan 71005, Taiwan; 6 Department of Anesthesiology, Tri-Service General Hospital and National Defense Medical Center, Taipei 11490, Taiwan; 7 Department of Food Science and Applied Biotechnology, National Chung Hsing University, Taichung 40227, Taiwan; mlhuhu@nchu.edu.tw; 8 Department of Senior Citizen Service Management, Chia Nan University of Pharmacy and Science, Tainan 71710, Taiwan

**Keywords:** vitamin C deficiency, postherpetic neuralgia, Leeds assessment of neuropathic symptoms and signs (LANSS) questionnaire, low intake, smoking, peptic ulcer disease

## Abstract

Vitamin C deficiency increases the risk of postherpetic neuralgia (PHN). In this cross-sectional study, the relationships among plasma vitamin C concentrations, pain and Leeds assessment of neuropathic symptoms and signs (LANSS) items were investigated during their first pain clinic visit of 120 PHN patients. The factors associated with vitamin C deficiency were determined. Independent predictors of vitamin C deficiency were presented as adjusted odds ratios (AOR) and 95% confidence intervals (CI). The patients had a high prevalence (52.5%) of vitamin C deficiency. Their plasma vitamin C concentrations were negatively associated with spontaneous pain and tingling, prickling or pins and needles sensation according to the LANSS questionnaire. Based on the receiver operator characteristic curve, the cutoffs for plasma vitamin C to predict moderate-to-severe and severe symptoms of sharp sensation were <7.05 and <5.68 mg/L, respectively. By comparison, the patients well-nourished with vitamin C had lower incidences of sharp sensations, sharp pain, and reddish skin. Multivariate analyses revealed that vitamin C deficiency was associated with the low intake of fruit/vegetables (AOR 2.66, 95% CI 1.09–6.48, *p* = 0.032), peptic ulcer disease (AOR 3.25, 95% CI 1.28–8.28, *p* = 0.014), and smoking (AOR 3.60, 95% CI 1.33–9.77, *p* = 0.010). Future studies are needed to substantiate these findings.

## 1. Introduction

Herpes zoster (HZ, shingles), which is caused by reactivated varicella-zoster viral (VZV) infection of the sensory ganglions and peripheral nerves, is characterized by its painful, blistering skin eruptions with dermatomal distribution [1]. The incidence of HZ ranged from 3 to 5 per 1000 person-years globally. Although most cases of HZ are self-limited, 5% to over 30% of HZ patients may continue to experience pain for months or even years after the resolution of the rash, particularly in the older adults [2]. Prolonged herpetic pain, also known as postherpetic neuralgia (PHN) [2], is a neuropathic pain syndrome presenting with heterogenous patterns of sensory dysfunction as well as spontaneous and stimuli-evoked pain [3]. Multiple mechanisms have been proposed for PHN [3], including dysregulation of *N*-methyl-D-aspartate (NMDA) receptors [4], dysfunction of Ca(v)3.2 T-channels [5,6] and chronic VZV-induced ganglionitis [7]. Because the risk of developing PHN increased sharply with age and comorbidities [8], the worldwide expansion of the elderly population has highlighted the urgency of the PHN issue.

Vitamin C (ascorbate) is an essential micronutrient for human health. Physiologically, vitamin C plays a vital role in a number of biological functions such as collagen synthesis, immune function, synthesis of catecholamines, opioid peptides and myelination as well as neuron protection [9,10]. Vitamin C has been shown to enhance synthesis of beta-endorphin and endomorphins [9,10,11], both of which have well-known analgesic effects, particularly for neuropathic pain [11,12,13]. Furthermore, vitamin C can reversibly inhibit Ca(v)3.2 T-channels [6,14], which regulate neuronal firing and synaptic transmission at dorsal horn synapses. Upregulation and increased activity of Ca(v)3.2 T-channels in the damaged dorsal root ganglion neurons contributes to neuropathic pain after peripheral nerve injury [15]. Evidence indicates that vitamin C exhibits analgesic properties. The authors and others have demonstrated that the prevalence of vitamin C deficiency in the patients with HZ and PHN is high and that vitamin C deficiency independently predicts the development of HZ and PHN [16,17,18,19,20,21]. Intravenous high-dose vitamin C decreased spontaneous pain effectively in PHN [18,22].

Spontaneous pain symptoms include tingling, prickling, pins and needles sensation, as well as electric shock, bursting, jumping, shooting, stabbing and burning pain [3]. The activation of specific receptors in the primary afferent fibers evokes pain, including myelinated A-delta fiber and unmyelinated C fiber. A-delta fibers evoke a rapid, sharp, prickling, tingling pain reaction [23] by releasing glutamate onto second-order neurons, while C fibers evoke a dull, pressing or burning sensation by releasing neuropeptide neurotransmitters [23]. Vitamin C is widely distributed in all of the body tissues. Its level is high in neurons and immune cells. The adults with low vitamin C levels are predisposed to HZ [17]. When the VZV became active in the cell bodies of dorsal root ganglion, the outbreak of HZ occurred. The infection unmasked the suboptimal levels of vitamin C because vitamin C boosted immune defense. Intriguingly, extracellular vitamin C at physiological levels can protect neurons from glutamate excitotoxicity induced by the activation of the NMDA receptors (glutamate-gated cation channels) [24]. Intracellular ascorbate protects neurons by scavenging reactive oxygen species (ROS) generated during glutamate-induced excitotoxicity [25]. Overall, vitamin C directly prevents excessive nerve stimulation caused by glutamate through scavenging ROS [25] and decreasing cell membrane levels of the NMDA receptor as well as inhibiting glutamate uptake via decreasing cell surface levels of the neuronal glutamate transporter (excitatory amino acid transporter 3) [26]. The authors therefore hypothesize that vitamin C deficiency may be associated with increased rapid, sharp sensations and increased sharp pain. The Leeds assessment of neuropathic symptoms and signs (LANSS) Pain Scale, which comprises seven items (five symptoms and two physical findings), is a questionnaire-based assessment tool for discriminating between neuropathic and nociceptive pain [27]. In this cross-sectional study, the relationships among vitamin C concentrations, spontaneous pain and the seven items of LANSS were investigated in 120 PHN patients.

The etiologies of vitamin C deficiency include low dietary intakes [17], increased requirements of vitamin C due to smoking [28] and lactation [29], decreased bioavailability of vitamin C resulting from peptic ulcer disease (PUD) [30,31], and convective/diffusive losses during dialysis as in chronic renal failure [32]. To the authors’ knowledge, no study has investigated the predictors associated with vitamin C deficiency defined by plasma concentrations (i.e., <6 mg/L or 34.1 *μ*mol/L) [33] in PHN patients. Therefore, factors including age, gender, low intake of fruits and vegetables, smoking and comorbidities were examined to identify the significant predictors associated with plasma vitamin C deficiencies in the clinical setting of PHN.

## 2. Materials and Methods

### 2.1. Study Site and Subjects

This study protocol complied with the Declaration of Helsinki. The Institutional Review Board Committee of Chi Mei Medical Center, Tainan, Taiwan approved this study. All subjects gave written informed consent prior to participation. This study was registered under UMIN Clinical Trials Registry (UMIN-CTR, http://www.umin.ac.jp/; Registration No.: R000013314, Trial ID: UMIN000011367).

A prospective, cross-sectional study was conducted at Chi Mei Medical Center’s pain clinic from January 2004 to December 2005. Subjects were eligible if aged 20–85 years, diagnosed with PHN (after ruling out other possible polyneuropathies) for at least 3 months but less than 2 years and having an average daily spontaneous pain score of at least 3 on an 11-point numeric rating pain scale (NRS) (0 being no pain and 10 being the worst pain imaginable) [18,34]. The PHN patients were interviewed according to a pre-designed questionnaire as well as received a plasma vitamin C survey thorough physical examinations during their first visit to our pain clinic [18,19]. The questionnaire included age, gender, height, weight, personal habits (i.e., smoking and intakes of fruits and vegetables), intensity of daily average spontaneous pain over the previous 24 h and brush-evoked pain, selected comorbidities and the LANSS questionnaire (Appendix A). The LANSS Pain Scale published in 2001 was the first screening tool for discriminating neuropathic from nociceptive pain [27]. Comorbidities including hypertension, diabetes mellitus, cancers, chronic obstructive pulmonary disease, PUD, hypercholesterolemia and dialysis with chronic kidney disease were selected because of their being reported to be associated with an increased risk of HZ and/or PHN [35,36,37,38,39]. Concurrent or pre-existing PUD was defined as having gastroendoscopy-proven PUD within one year prior to the outbreaks of HZ [37,40]. Hypercholesterolemia was defined as having a total cholesterol level of 240 mg/dL or higher [41]. Low intake of fruits and vegetables before outbreaks of shingles was defined as having ≤3 servings per day [17]. Heavy smoking was defined as >1 pack of cigarettes per day [42].

### 2.2. Measurement of Plasma Vitamin C Concentrations

Blood samples from the patients in lithium heparin vacutainers after overnight fasting were transported to the laboratory in a light-excluding container and stored for no more than 6 h at 4°C before centrifugation. Plasma vitamin C concentrations were measured by the automated enzymatic method as described in the previous articles [18,19].

### 2.3. Evaluation of Spontaneous Pain and Items in the LANSS Questionnaire

The spontaneous pain intensity was measured on the 11-point NRS (0–10) [18]. The severity of three symptoms and one physical finding in the LANSS questionnaire including tingling, prickling or pins and needles sensations, sudden electric shocks, bursting, jumping pain, burning pain as well as allodynia was also assessed on the 11-point NRS (0–10). A different skin aspect, abnormally sensitive to touch and altered pin-prick threshold were determined and recorded by the physician.

### 2.4. Statistical Analysis

All statistical analyses were performed using the SAS software package, version 9.3 (SAS Institute, Cary, NC, USA), and the statistical significance level was set at *p* < 0.05 (two-sided). Either Chi-square or Fisher’s exact test was used to differentiate the statistical differences in categorical variables. Student’s t-test was used to test the differences in numerical variables.

The normality of variables was examined with the Kolmogorov–Smirnov test. Pearson’s or Spearman’s correlation was performed to test the significance of association between the plasma vitamin C concentrations and the severity of spontaneous pain/items in the LANSS questionnaire wherever appropriate. The correlation between plasma vitamin C concentrations and the severity of spontaneous pain/items in the LANSS questionnaire was considered to be clinically significant if the rho > 0.3 [19]. To identify the optimal cutoff point for predicting mild symptoms of tingling, prickling or pins and needles sensation (i.e., NRS < 4) [43], a receiver operating characteristic (ROC) curve was plotted. The optimal cutoff value was determined with the Youden’s index via maximizing the point on the ROC curve furthest from the line of equality. The area under the ROC curve (AUC) was used to measure the diagnostic ability of the plasma vitamin C concentration. For identifying the optimal cutoff point for predicting severe symptoms of tingling, prickling or pins and needles sensation (i.e., NRS 7–10) [43], a ROC curve was plotted. The optimal cutoff value was determined with the Youden’s index via maximizing the point on the ROC curve furthest from the line of equality. The AUC was used to measure the diagnostic ability of the plasma vitamin C concentration. Furthermore, the proportions of items in the LANSS questionnaire between patients with plasma vitamin C concentrations ≤ the cutoff point and those with levels > the cutoff point were compared to identify the associations between the plasma vitamin C concentration and the various symptoms/physical findings. A *p* value of <0.05 was considered statistically significant.

The univariate logistic regression models were adopted to identify the independent predictors of ascorbate deficiency including age, gender, comorbidities and personal habits. Multivariate logistic regression with an entry criterion of *p* < 0.2 was used to determine the risk factors and to estimate adjusted odds ratios (OR) and their 95% confidence interval (CI). Multivariate logistic regression with backward eliminating procedure was applied to the determination of the final model with an exit criterion set as *p* > 0.05.

## 3. Results

**Figure 1:**
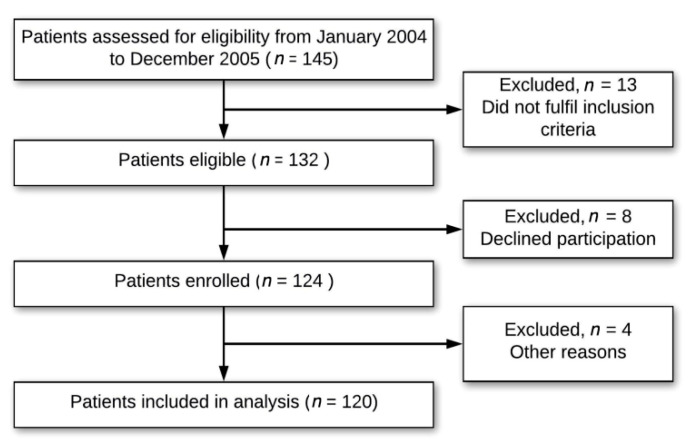
Clinical study flowchart.

Figure 1 shows the flow chart of the study, which was conducted in the years preceding the licensure of the zoster vaccine by the US Food and Drug Administration (FDA) in 2006 [1]. Baseline characteristics of the 120 PHN patients are presented in Table 1. None of the patients received vaccinations against HZ prior to the development of HZ. Only 19 (15.8%) PHN patients had well-nourished levels of plasma vitamin C. A high prevalence (52.5%) of vitamin C deficiency (<6 mg/L) was found in the PHN patients.

### 3.1. Primary Outcomes: Correlations between Spontaneous Pain/Items in the LANSS and Plasma Vitamin C in PHN Patients

As shown in Table 2, the patients’ plasma vitamin C concentrations were negatively correlated with spontaneous pain (Spearman correlation coefficient: −0.420, *p* < 0.001) and with tingling, prickling or pins and needles sensation (Spearman correlation coefficient: −0.449, *p* < 0.001). The other Spearman correlation coefficients were ≤ 0.3, indicating no clinical significance.

### 3.2. Secondary Outcomes: The Cutoff for Plasma Vitamin C Concentrations to Predict Tingling, Prickling or Pins and Needles Sensation

**Figure 2:**
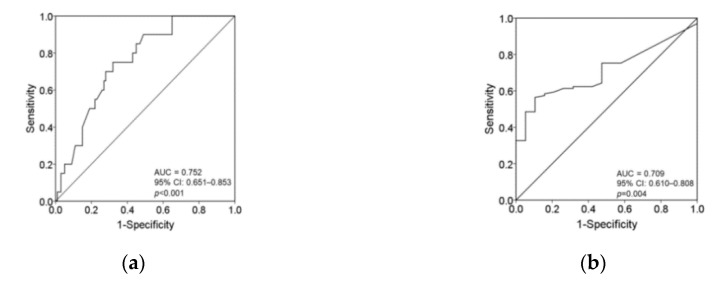
(**a**) The area under the receiver operating characteristic curve for vitamin C concentration associated with an increased incidence of moderate-to-severe symptoms of tingling, prickling or pins and needles sensation (i.e., NRS ≥ 4); (**b**) The area under the receiver operating characteristic curve for vitamin C concentration to predict severe tingling, prickling or pins and needles sensation (i.e., NRS ≥ 7).

The cutoff value for plasma vitamin C levels associated with an increased incidence of moderate-to-severe symptoms of tingling, prickling or pins and needles sensation (i.e., NRS ≥ 4) was <7.05 mg/L (sensitivity 75.0%; specificity 68.0%). The AUC was 0.752 (95% CI 0.651–0.853; *p* < 0.001). The cutoff value for serum vitamin C levels to predict severe symptoms of tingling, prickling or pins and needles sensation (i.e., NRS ≥ 7) was <5.68 mg/L (sensitivity 56.4%; specificity 89.5%). The AUC was 0.709 (95% CI 0.610–0.808; *p* = 0.004) (Figure 2a,b).

### 3.3. The Proportions of Items in the LANSS Questionnaire in Patients with Different Plasma Vitamin C

Of the 120 PHN patients, 93 (77.5%) had a score greater or equal to 12 in the LANSS questionnaire.

The proportions of positive items in the LANSS questionnaire were compared between patients with vitamin C concentrations ≥ the cutoff value and those with vitamin C concentrations < the cutoff value. Based on the cutoff for mild symptoms (NRS < 4) of tingling, prickling or pins and needles sensation, the patients with vitamin C ≥ 7.05 mg/L had a lower prevalence (61.7%) of tingling, prickling or pins and needles sensations compared with that (97.3%) in those with vitamin C < 7.05 mg/L (*p* < 0.001). Likewise, based on the cutoff for severe symptoms (NRS ≥ 7) of tingling, prickling or pins and needles sensation, the patients with vitamin C < 5.68 mg/L had a higher prevalence (96.7%) of tingling, prickling or pins and needles sensations compared with that (74.6%) in those with vitamin C ≥ 5.68 mg/L (*p* < 0.001). No significant differences were noted in the proportions of positive scoring for the other items between the high and low vitamin C concentration groups using either cutoff value (Table 3).

Based on the cutoff for vitamin C deficiency (<6.0 mg/L), the patients with vitamin C deficiency had a greater proportion (96.8%) of tingling, prickling or pins and needles sensations compared to that (68.4%) in those with vitamin C ≥ 6.0 mg/L (*p* < 0.001). Based on the cutoff for well-nourished levels of vitamin C (≥10 mg/L), patients with well-nourished levels had lower proportions of tingling, prickling or pins and needles sensations, a different skin aspect in the painful areas, sudden electric shocks, bursting or jumping pain (57.9%, 42.1%, 31.6%, respectively) compared with those in patients with vitamin C < 10 mg/L(88.1%, 68.3%, 56.4%; *p* < 0.001, 0.029, 0.047), respectively (Table 4).

### 3.4. Factors Associated with Plasma Vitamin C deficiency

Multivariate logistic analysis revealed that the low intake of fruits and vegetables before outbreaks of HZ (adjusted OR 2.66, 95% CI: 1.09–6.48, *p* = 0.032), PUD (adjusted OR 3.25, 95% CI: 1.28–8.28, *p* = 0.014) and smoking (adjusted OR 3.60, 95% CI: 1.33–9.77; *p* = 0.010) were significantly associated with plasma vitamin C deficiency in PHN patients (Table 5).

## 4. Discussion

The present study revealed that patients with PHN had a high prevalence of vitamin C deficiency. PHN is neuropathic pain that can be spontaneous (stimulus-independent) and/or stimulus-dependent. An 11-point NRS is a common tool for the evaluation of pain severity [18,34]. The intensity of spontaneous pain based on the 11-point NRS was found to be negatively correlated with plasma vitamin C concentrations in the present study. The finding is consistent with the reports by the authors and others [18,19,21,22]. However, one-dimensional scales are inadequate for the assessment of neuropathic pain [44]. Spontaneous pain symptoms include tingling, prickling, pins and needles sensation, as well as electric shock, bursting, jumping, shooting, stabbing and burning pain. A symptom-based approach to the assessment of painful neuropathy is suggested to be useful for identifying the underlying mechanisms [45]. The current study demonstrated that, among items in the LANSS Pain Scale, the intensity of sharp tingling, prickling or pins and needles sensations evaluated on the 11-point NRS was inversely associated with the plasma vitamin C concentrations. Although the present study did not explore the underlying mechanism, the authors nevertheless provide some possible explanations as follows: First, the A-delta fibers from first-order neurons evoke a rapid, sharp, prickling, tingling pain reaction by releasing glutamate onto the second-order neurons [23]. Vitamin C decreases glutamate-stimulated NMDA receptor activity and inhibits the binding of glutamate to NMDA receptors through decreasing the surface expression of NMDA receptors [26]. Hence, vitamin C can directly prevent excessive glutamate-induced nerve stimulation [26]. Second, vitamin C supplementation has been reported to attenuate neuronal damage through decreasing glutamate-induced over-activation of glutamate receptors in an animal study [46]. Taken together, PHN patients with vitamin C deficiency may experience sharp sensation and sudden sharp pain due to a reduced inhibition of glutamate binding onto the NMDA receptors. Third, a previous study based on the Douleur Neuropathique 4 questionnaire discovered that hypovitaminosis D was associated with increased spontaneous and brush-evoked pain. The patients with vitamin D <67.0 nmol/L were more likely to suffer from spontaneous painful cold [47]. It seems that there are associations between nutritional deficiency and sensory symptoms/signs. The findings support that a symptomatic approach based on neuropathic pain questionnaires can help elucidate the mechanisms underlying specific symptoms and signs [45].

Based on the ROC curve, the cutoff concentrations of plasma vitamin C for the prediction of moderate-to-severe and severe symptoms of tingling, prickling or pins and needles sensation were < 7.05 mg/L and <5.68 mg/L, respectively. Interestingly, the patients well-nourished with vitamin C (≥10 mg/L) not only had a lower prevalence of tingling, prickling or pins and needles sensations but also experienced less sharp pain such as sudden electric shocks, bursting or jumping pain compared with those in the patients with vitamin C <10 mg/L. Tingling, prickling or pins and needles sensations are sharp unpleasant sensations, while sudden electric shocks, bursting or jumping pain are sharp pain. The findings imply that different thresholds of plasma vitamin C exist for sharp sensation and sharp pain. In cell models, glutamate-induced neuronal excitotoxicity is highly dose-dependent [48]. Furthermore, the antinociceptive effects of vitamin C have been shown to be dose-dependent after peripheral nerve injury [49]. These findings support the proposal that the therapeutic level of plasma vitamin C for sharp pain may be higher than that for treating sharp sensation. A number of previous studies have shown that intravenous vitamin C 7.5 g can reduce PHN, but a total dosage of intravenous vitamin C ≥ 35 g may be required for relieving acute herpetic pain [18,21,50,51]. Kim et al. (2016) reported that intravenous administration of vitamin C 15 g did not relieve acute herpetic pain, but it was effective for reducing the incidence of PHN [52]. Therapeutic dosages for acute herpetic pain seem to be higher than those for chronic herpetic pain. More studies are needed to determine the recommended dosages of intravenous vitamin C for the treatment of acute and chronic herpetic pain. Theoretically, plasma vitamin C concentrations may be inversely correlated with the intensity of sudden electric shocks, bursting, jumping pain. However, the Spearman correlation coefficient between plasma vitamin C concentrations and sudden sharp pain (electric shocks, bursting, jumping pain) in the present study was ≤0.3, indicating no clinical significance. Possibly, either too few patients with sudden electric shocks, bursting or jumping pain or too few patients well-nourished with vitamin C being enrolled in the present small-scale study may lead to the false negative correlation [53]. More studies are required to explore the link between the various sensory signs/symptoms and the pathophysiological mechanisms involving different types of nutritional deficiency to open up new avenues for more effective and specific mechanism-based treatments.

In the present study, there were 93 patients with a score greater or equal to 12 in the LANSS questionnaire among the 120 PHN patients. The overall accuracy was 77.5% (93/120). The results indicate a good correlation between the clinical diagnosis and the LANSS questionnaire scores with cut-offvalues ≥ 12. However, clinical diagnosis by the physician is still necessary [54]. From the LANSS questionnaire, we observed that the patients well-nourished with vitamin C had a lower incidence of reddish skin in the painful areas than that in those with vitamin C deficiency. Local reddish skin indicates hypoactive sympathetic activity. The composition of most peripheral nerves is mixed, consisting of motor, sensory and autonomic nerve fibers. Postganglionic sympathetic fibers release norepinephrine as their transmitter to act on target tissues [55]. Norepinephrine synthesis is ascorbate-dependent [56,57]. Some explanations are made for the link between the increased incidence of reddish skin and vitamin C deficiency. First, vitamin C enhances tyrosine hydroxylase expression. Second, ascorbate helps to maintain the activity of the enzyme tyrosine hydroxylase by recycling its essential co-factor—tetrahydrobiopterin. Third, vitamin C acts as a cofactor for dopamine β-hydroxylase to hydroxylate dopamine to form norepinephrine by directly contributing an electron to dopamine β-hydroxylase in neurosecretory vesicles [56,57]. Therefore, the PHN patients with plasma vitamin C<10 mg/L may have a greater incidence of reddish skin due to hypoactive sympathetic activity associated with reduced norepinephrine formation. However, the Spearman correlation coefficient between plasma vitamin C concentrations and the incidence of different dermatologic conditions (i.e., reddish skin) in the present study was −0.250, indicating no clinical significance. The false-negative results may be attributed to the small-number of cases with different dermatologic conditions. Optimization of plasma vitamin C to well-nourished levels by vitamin C supplementation may potentially reduce reddish skin in the painful areas.

Vitamin C deficiency was significantly associated with the low intake of fruits and vegetables, PUD and smoking in multivariate analysis. The results are consistent with those of previous research [17,28,30] reporting that a low vitamin C intake, PUD and smoking are common etiologies of vitamin C deficiency. Vitamin C is synthesized from glucose via the glucuronic acid pathway in animals. During the evolutionary process, the biosynthetic capacity of vitamin C in humans was lost because of a genetic defect that led to a failure of synthesizing L-gulonolactone oxidase, which is the terminal enzyme in vitamin C biosynthesis in animals [58]. Thus, vitamin C is an essential micronutrient for human health and totally dependent on dietary intake in humans. The risk of HZ was found to be strongly associated with low fruit intakes in a case-control study [17]. Consistent with the finding of that research, the low intake of fruits and vegetables independently predicted vitamin C deficiency in our PHN patients. PUD, which has been found to be a risk factor of HZ and PHN [20,36,40], was also identified as another predictor of vitamin C deficiency in the patients with PHN in the current study. Two major risk factors of PUD include *Helicobacter pylori* (*H. pylori*) infection and the use of nonsteroidal anti-inflammatory drugs (NSAIDs). The impairment of systemic bioavailability of vitamin C in patients with *H. pylori* infection was not related to diet [59]. In vitro, NSAIDs inhibit cellular vitamin C uptake in a dose-dependent and non-competitive manner [60]. In humans, the use of NSAIDs has been shown to reduce plasma vitamin C levels [31]. Furthermore, medications like proton pump inhibitors decreased plasma vitamin C levels due to a reduced intestinal absorption [61]. Overall, vitamin C deficiency is often concomitantly present in patients with *H. pylori*-infected and NSAID-associated PUD. These findings provide a reasonable basis for PUD to be identified as a risk factor for vitamin C deficiency in PHN patients. Parruti G. et al. (2010) identified smoking as an independent predictor of HZ and PHN in a prospective cohort study [62]. In particular, smoking was significantly associated with the intensity and persistence of herpetic pain [62]. A reduced plasma vitamin C concentration is commonly observed among smokers. Out of the 31 current smokers among our PHN patients, approximately two-thirds (*n* = 21, 67.7%) were heavy smokers (>1 pack a day [42]). Tobacco smoke containing large numbers of radicals burdens the antioxidant defense and, thus, lowers plasma antioxidant levels, in particular vitamin C [28,63]. Increased vitamin C degradation by the oxidative stress reduces plasma vitamin C concentration in smokers with PHN [62]. The results highlight the relationships among smoking, vitamin C deficiency and the risk of HZ and PHN. Taken together, low vitamin C intakes, smoking and PUD are predictors for vitamin C deficiency in patients with PHN. According to the findings that the intensity of spontaneous pain as well as sharp tingling, prickling or pins and needles sensations were inversely associated with plasma vitamin C concentrations, increasing intakes of fruit and vegetables, quitting smoking and treating PUD are suggested to facilitate the management of PHN.

There were some limitations in this study. First, the diagnosis of PHN was made clinically based on the symptoms and signs as well as the disease course without serological confirmation. However, dermatomal skin eruptions are characteristic of the disease. Besides, there is a high agreement (92%) between the clinical diagnosis of HZ and the result of polymerase chain reaction assay [64]. Second, the present study did not compare the vitamin C levels of PHN patients with those of HZ patients who did not go on to develop PHN. Third, aged adults in poor vitamin C status have a significantly increased prevalence of depression symptoms [65], which may be associated with an increase the risk and severity of HZ [66]. However, patients with major depressive disorders were not included in the present study. Fourth, chronic kidney disease with dialysis is a risk factor of vitamin C deficiency [32], no eligible patients were enrolled in this study, so that whether the findings are applicable to this patient population remained unclear. Fifth, although ethnicity is an independent predictor of the occurrence of HZ and PHN [67], only Taiwanese were recruited in the current study. Therefore, further large-scale studies on other ethnic groups are warranted to support the findings. Sixth, the low vitamin C may just reflect a low overall nutrition status of the PHN patients. No adjustment for other nutrition factors such as vitamin B12 was a limitation of this study. Vitamin B12 enhances the myelination process and decreases ectopic nerve firing. A recent systemic review revealed that vitamin B12 treatment can help to relieve PHN [68].

## 5. Conclusions

Patients with postherpetic neuralgia had a high prevalence of vitamin C deficiency. Plasma vitamin C concentrations were negatively associated with spontaneous pain and sharp tingling, prickling or pins and needles sensations. The cutoff plasma vitamin C concentrations for predicting severe tingling, prickling or pins and needles sensation as well as moderate-to-severe symptoms were <5.68 mg/L and <7.05 mg/L, respectively. The patients well-nourished with vitamin C (>10 mg/L) also had lower incidences of sharp sensations, sharp pain and reddish skin. Low intake of fruits and vegetables, peptic ulcer disease and smoking are predictors of vitamin C deficiency in patients with postherpetic neuralgia. Taken together, screening and optimization of plasma vitamin C levels are suggested in the management of postherpetic neuralgia presenting with sharp sensation, sharp pain [56] and reddish skin in the affected area, in particular in patients with low intake of fruits and vegetables, peptic ulcer disease and smoking. Future studies are warranted to substantiate the associations between the symptoms of postherpetic neuralgia and plasma concentrations of other nutrients such as vitamin B12. In addition, other possible confounding factors such as kidney disease and patient ethnicity should be taken into consideration.

## Figures and Tables

**Table 1:** Baseline characteristics of 120 patients suffering from PHN.

Parameters	Mean (SD)
Age, mean (SD) (years)	66.45 (11.34)
Body height, mean (SD) (cm)	158.67 (11.42)
Body weight, mean (SD) (kg)	55.99 (11.86)
Male, *n* (%)	63 (52.5%)
Duration of pain, mean (SD) (months)	8.55 (7.50)
Plasma concentrations of vitamin C (6–15 mg/L)	
mean (SD) (mg/L)	6.34 (3.80)
Well-nourished (≥10 mg/L; 56.8 *μ*mol/L), *n* (%)	19 (15.8%)
Adequate (6–10 mg/L; 34.1–56.8 *μ*mol/L), *n* (%)	38 (31.7%)
Deficiency (<6 mg/L; 34.1 *μ*mol/L), *n* (%)	63 (52.5%)
Comorbidities/habits (*n*, %)	
Hypertension	47 (39.2%)
Diabetes mellitus	31 (25.8%)
Peptic ulcer disease	37 (30.8%)
Cancers	12 (10.0%)
COPD	15 (12.5%)
Chronic kidney disease with dialysis	0 (0%) ^a^
Hypercholesterolemia	13 (10.8%)
Smoking (Male: 31; Female: 0; heavy smokers ^b^: 21)	31 (25.8%)
Alcohol intake	5 (4.2%)
Low intake of fruits and vegetables ^c^	46 (38.3%)

COPD: chronic obstructive pulmonary disease; SD: standard deviation; NRS: 11-point numeric rating pain scale (0–10); PHN: postherpetic neuralgia. ^a^ During the study period, two PHN patients suffering from chronic kidney disease with dialysis met the inclusion criteria but declined to join the study. ^b^ Heavy smoking was defined as >1 pack of cigarettes per day. ^c^ Low intake of fruits and vegetables was defined as having ≤3 servings per day.

**Table 2:** Correlations between plasma vitamin C concentrations and spontaneous pain/items in the LANSS questionnaire among PHN patients.

	Spearman Correlation Coefficient	*p*
Plasma vitamin C concentrations vs. Spontaneous pain (NRS 0–10)	−0.420 *	<0.001
vs. Items in the LANSS questionnaire		
Tingling, prickling or pins and needles sensation (NRS 0–10)	−0.449 *	<0.001
A different skin aspect (Yes: 1; No: 0)	−0.250	0.007
Abnormally sensitive to touch (Yes: 1; No: 0)	−0.231	0.011
Sudden electric shocks, bursting, jumping pain (NRS 0–10)	−0.104	0.265
Burning pain (NRS 0–10)	−0.173	0.058
Allodynia (NRS 0–10)	−0.139	0.131
Altered pin-prick threshold (Yes: 1; No: 0)	−0.113	0.218

LANSS: Leeds assessment of neuropathic symptoms and signs. NRS: 11-point numeric rating pain scale (0–10); PHN: postherpetic neuralgia. * Spearman correlation coefficients indicate clinical significance if the value is greater than 0.3.

**Table 3:** The proportions of positive items in the LANSS questionnaire among patients with vitamin C concentrations ≥ the cutoff value vs. < the cutoff value.

Cutoff for Plasma Vitamin C Concentrations	≥7.05 mg/L (*n* = 47)	<7.05 mg/L (*n* = 73)	*p*	≥5.68 mg/L (*n* = 57)	<5.68 mg/L (*n* = 63)	*p*
Tingling, prickling or pins and needles sensations, *n* (%)	29 (61.7)	71 (97.3)	<0.001	44 (74.6)	59 (96.7)	<0.001
A different skin aspect in the painful areas, *n* (%)	26 (55.3)	51 (69.9)	0.105	34 (57.6)	43 (70.5)	0.142
Abnormally sensitive to touch in the painful area, *n* (%)	18 (38.3)	36 (49.3)	0.236	23 (39.0)	31 (50.8)	0.193
Sudden electric shocks, bursting or jumping pain, *n* (%)	24 (51.1)	39 (48.9)	0.800	32 (54.2)	31 (50.8)	0.708
Burning pain, *n* (%)	9 (19.1)	22 (30.1)	0.180	13 (22.0)	18 (29.5)	0.350
Allodynia in painful area, *n* (%)	23 (48.9)	45 (61.6)	0.170	29 (49.2)	39 (63.9)	0.102
Altered pin-prick threshold, *n* (%)	20 (42.6)	21 (28.8)	0.120	24 (40.7)	17 (27.9)	0.139

*n*: number; LANSS: Leeds assessment of neuropathic symptoms and signs.

**Table 4:** The proportions of positive items in the LANSS questionnaire among patients with vitamin C concentrations ≥ the cutoff value vs. < the cutoff value.

	Well-Nourished			Deficient		
Cutoff for Plasma Vitamin C Concentrations	≥10 mg/L (*n* = 19)	<10 mg/L (*n* = 101)	*p*	≥6.0 mg/L (*n* = 57)	<6.0 mg/L (*n* = 63)	
Tingling, prickling or pins and needles sensations, *n* (%)	11 (57.9)	89 (88.1)	<0.001	39 (68.4)	61 (96.8)	<0.001
A different skin aspect in the painful areas, *n* (%)	8 (42.1)	69 (68.3)	0.029	33 (57.9)	44 (69.8)	0.173
Abnormally sensitive to touch in the painful area, *n* (%)	6 (31.6)	48 (47.5)	0.200	22 (38.6)	32 (50.8)	0.180
Sudden electric shocks, bursting or jumping pain, *n* (%)	6 (31.6)	57 (56.4)	0.047	31 (54.4)	32 (50.8)	0.694
Burning pain, *n* (%)	2 (10.5)	29 (28.7)	0.097	12 (21.1)	19 (30.2)	0.255
Allodynia in painful area, *n* (%)	7 (36.8)	61 (60.4)	0.057	28 (49.1)	40 (63.5)	0.113
Altered pin-prick threshold, *n* (%)	10 (52.6)	31 (30.7)	0.064	24 (42.1)	17 (27.0)	0.081

*n*: number;LANSS: Leeds assessment of neuropathic symptoms and signs.

**Table 5:** Multivariate logistic analysis for plasma vitamin C deficiency in 120 PHN patients.

	Vitamin C Deficiency (<6 mg/L) (*n* = 63, 52.5%)			
Variables	Crude Odds Ratio (95% CI)	*p* ^b^	Adjusted Odds Ratio (95% CI)	*p* ^b^
Gender (male vs. female)	2.23 (1.08, 4.64)	0.030 *		
Age (≥70 vs. <70 years old)	0.89 (0.43, 1.84)	0.761		
Hypertension	2.48 (1.16, 5.31)	0.018 *		
Diabetes mellitus	1.95 (0.84, 4.53)	0.120		
Peptic ulcer disease	5.95 (2.60, 13.61)	<0.001 *	3.25 (1.28–8.28)	0.014 *
Cancer	3.0 (0.77, 11.69)	0.100		
COPD	1.42 (0.47, 4.26)	0.534		
Hypercholesterolemia	1.06 (0.33, 3.37)	0.918		
Smoking	10.65 (4.30, 26.37)	<0.001 *	3.60 (1.33–9.77)	0.010 *
Alcohol intake	3.80 (0.41, 35.01)	0.208		
Low intake of fruits and vegetables before outbreaks of herpes zoster ^a^	11.61 (4.53, 29.72)	<0.001 *	2.66 (1.09–6.48)	0.032 *

COPD: chronic obstructive pulmonary disease; PHN: postherpetic neuralgia; CI: confidence interval. ^a^ Low intake of fruits and vegetables was defined as having *≤*3 servings per day. ^b^ The *p* value (determined using logistic multivariate regression) represents the significance level for having certain medical conditions and low vitamin C intakes in patients with vitamin C deficiency compared to patients with adequate vitamin C. * p< 0.05, considered statistically significant.

## Data Availability

Anonymized data not published within this article will be made available and shared by request from any qualified investigator.

## Abbreviations

AUC	area under the ROC curve
*H. pylori*	*Helicobacter pylori*
HZ	herpes zoster
LANSS	Leeds assessment of neuropathic symptoms and signs Pain Scale
NMDA	*N*-methyl-D-aspartate
NRS	numeric rating pain scale
NSAIDs	nonsteroidal anti-inflammatory drugs
PHN	postherpetic neuralgia
PUD	peptic ulcer disease
ROS	reactive oxygen species
VZV	varicella-zoster virus

## Appendix A

**Chi Mei Medical Center Pain Clinic**
**First Visit Questionnaire**

First visit date: (dd/mm/yy)

Name: Medical record number: Birthday:

(1) Basic information:

Smoking: No/Yes, years; package/day; quitted for years

Drinking: No/Yes, year; (Frequency: every day, days a week; bottles/time; totally mL; Beer/sorghum/Whiskey)

Betel nut: No/Yes, for years; pack/day; quitted for years

Low intake of fruits and vegetables (≤3 servings per day): low ______ vs. normal ______

(2) Past medical history:

Hypertension: No/Yes, for years (Medication:) BP: HR:

Diabetes: No/Yes, for years (Medication:/RI year).

Cancer: No/Yes. (years ago)- left/right (breast/lung/kidney)/pancreas/colon/___________

 Treatment: Surgery/Chemotherapy/Radiotherapy/Others: ___________

Peptic ulcers: No/Yes, for years (post treatment-years ago)

 *Helicobacter pylori*: No/Yes, for years

COPD: No/Yes.; Hypercholesterolemia: No/Yes

Autoimmune diseases (SLE, RA, Sjogren’s syndrome): No/Yes, for years

Hepatitis B/C carrier; liver cirrhosis: No/Yes;

Chronic kidney disease, hemodialysis/PD: No/Yes, for years (weekdays 1,3,5/2,4,6)

Other diseases: Hyperuricemia: No/Yes.; Glucose-6-phosphate dehydrogenase (G6PD) deficiency: No/Yes.; Stroke: No/Yes (times, years ago)

Surgery history (site___________/time ___________/hospital ___________):

Special medications: steroid, drug abuse, ___________

Drug allergy: No/Yes, (Drug:; Symptoms: rash//shock)

Supplementation: No/Yes, Regular/Irregular (multivitamins; vitamin C mg/day; calcium mg/day)

(3) Family history

Father: Diabetes/Hypertension/Stroke/Tumor

Mother: Diabetes/Hypertension/Stroke/Tumor

(4) History of herpes zoster

Sites: face/neck and shoulder/upper extremity/trunk/sacral/lower extremity

Nerve roots: left/right; C/T/L/S _______________________

Date of shingles outbreak: __________________________

Duration (from the zoster onset to the first visit at our pain clinic): ______ weeks/months

Referral to our pain clinic: due to (A); (B); (C) both_______

Poor response to treatment

Intolerable side effects (dizziness/nausea and vomiting/constipation/___________)

Have you taken antiviral drugs? No/Yes, for days/Don’t know.

Have you taken morphine? No/Yes, dosage:

Have you received nerve blocks? No/Yes ___________

(5) NRS scales on the previous 24 h

 (5–1) NRS scales (0–10) of average spontaneous pain:

 (5–2) NRS scales (0–10) of the worst spontaneous pain:

 (5–3) NRS scales (0–10) of brush-evoked pain:

(6) LANSS Questionnaire—Total points: (≥12)

1. Does the pain produce unpleasant sensations such as tingling, prickling or pins and needles?	Yes (5) No (0)
2. Is there a different skin aspect in the painful areas, i.e., skin redder than usual or appearing mottled?	Yes (5) No (0)
3. Does stroking the skin in the painful area or wearing tight clothing items produce unpleasant sensations?	Yes (3) No (0)
4. Do you experience any sensations like electric shocks, bursting or jumping corresponding to painful episodes, i.e., unexplained bursts of pain?	Yes (2) No (0)
5. Do you experience burning sensations in the painful areas or a sudden temperature change?	Yes (1) No (0)
6. Result from stroking the nonpainful area and the described painful area with cotton wool:	Allodynia in painful area (5) Normal sensations in both areas (0)
7. Result to touching (pinprick) both areas with a 23-gauge needle:	Altered PPT in the painful area (3) Equal sensations in both areas (0)
